# A Drd1-cre mouse line with nucleus accumbens gene dysregulation exhibits blunted fentanyl seeking

**DOI:** 10.1038/s41386-025-02116-0

**Published:** 2025-05-02

**Authors:** Annalisa Montemarano, Logan D. Fox, Farrah A. Alkhaleel, Alexandria E. Ostman, Hajra Sohail, Samiksha Pandey, Laura B. Murdaugh, Megan E. Fox

**Affiliations:** 1 Department of Anesthesiology and Perioperative Medicine, Pennsylvania State University College of Medicine, Hershey, PA USA; 2 Department of Neuroscience and Experimental Therapeutics, Pennsylvania State University College of Medicine, Hershey, PA USA

**Keywords:** Motivation, Reward, Genetics of the nervous system

## Abstract

The synthetic opioid fentanyl remains abundant in the illicit drug supply, contributing to tens of thousands of overdose deaths every year. Despite this, the neurobiological effects of fentanyl use remain largely understudied. The nucleus accumbens (NAc) is a central locus promoting persistent drug use and relapse, largely dependent on activity of dopamine D1 receptors. NAc D1 receptor-expressing medium spiny neurons (D1-MSNs) undergo molecular and physiological neuroadaptations in response to chronic fentanyl that may promote relapse. Here, we obtained Drd1-cre^120Mxu^ mice to investigate D1-dependent mechanisms of fentanyl relapse. We serendipitously discovered this mouse line has reduced fentanyl seeking, despite similar intravenous fentanyl self-administration, similar sucrose self-administration and seeking, and greater fentanyl-induced locomotion compared to wildtype counterparts. We found drug-naïve Drd1-cre^120Mxu^ mice have elevated D1 receptor expression in NAc and increased sensitivity to the D1 receptor agonist SKF-38393. After fentanyl self-administration, Drd1-cre^120Mxu^ mice exhibit divergent expression of MSN markers, opioid receptors, glutamate receptor subunits, and TrkB which may underly their blunted fentanyl seeking. Finally, we show fentanyl-related behavior is unaltered by chemogenetic manipulation of NAc core D1-MSNs in Drd1-cre^120Mxu^ mice. Conversely, chemogenetic stimulation of ventral mesencephalon-projecting NAc core MSNs (putative D1-MSNs) in wildtype mice recapitulated the blunted fentanyl seeking of Drd1-cre^120Mxu^ mice, supporting a role for aberrant D1-MSN signaling in this behavior. Together, our data uncover alterations in NAc gene expression and function with implications for susceptibility and resistance to developing fentanyl use disorder.

## Introduction

Opioid use disorder (OUD) is a chronic, relapsing condition characterized by uncontrollable opioid use and craving despite negative consequences, and the development of tolerance and withdrawal [1]. In 2022, over 9 million adults in the U.S. required OUD treatment [2], and over 80,000 individuals died from fatal overdose involving opioids [3]. A massive contributor to this epidemic is the current abundance of fentanyl in the illicit drug supply [4]. Fentanyl is a potent synthetic opioid that possesses unique pharmacological properties compared to other opioids: it has high lipid solubility that allows for rapid passage through the blood brain barrier [5], unique binding interactions with the mu opioid receptor [6], and higher selectivity for mu over kappa and delta opioid receptors as compared to morphine [7]. Fentanyl exposure is associated with poorer treatment outcomes for OUD [8], necessitating novel treatment approaches to cases of OUD involving fentanyl.

The vicious cycle of OUD, in which an abstinence-induced negative affective state promotes persistent relapse, is thought to be driven by neural circuitry centered around the nucleus accumbens (NAc) [1, 9]. The NAc, located in the ventral striatum, is primarily comprised of GABAergic medium spiny neurons (MSNs) that are divided into two subtypes depending on whether they express *Drd1* (encoding dopamine D1 receptor; D1-MSNs) or *Drd2* (encoding dopamine D2 receptor; D2-MSNs). The MSN subtypes can be further identified by co-expression of additional markers: D1-MSNs co-express *Chrm4* (muscarinic acetylcholine receptor M4), *Pdyn* (preprodynorphin), and *Tac1* (preprotachkykinin-1); D2-MSNs co-express *Adora2a* (adenosine A_2A_ receptor), *Gpr6* (G protein-coupled receptor 6), and *Penk* (preproenkephalin) [10, 11]. D1- and D2-MSNs have distinct projection targets [11–13] that typically drive opponent processes in reward behavior, where D1-MSN activity is classically considered “pro-reward” while D2-MSN activity is “anti-reward” [14–21], although there are numerous exceptions [22–29].

NAc MSNs undergo extensive physiological [30–42] and transcriptional [43–51] adaptations following opioid abstinence that promote negative affect during withdrawal or increase relapse to drug seeking. However, neuroadaptations vary across opioid class and MSN subtype. Our prior work demonstrates homecage oral fentanyl produces adaptations specifically in D1- but not D2-MSNs that underly negative affect during abstinence [52]. In contrast, intravenous heroin self-administration produces similar adaptations to both D1- and D2-MSNs that promote drug seeking behavior [53, 54]. Whether intravenous fentanyl self-administration produces subtype-similar, or subtype-specific neuroadaptations remains unknown.

To further understand how NAc D1-MSNs influence intravenous fentanyl self-administration and relapse to fentanyl seeking, we obtained a Drd1-cre mouse line deposited to the Jackson Laboratory [55] that has been used by several other groups [56–70]. First, we characterized baseline fentanyl self-administration and seeking behavior, and serendipitously discovered that the Drd1-cre^120Mxu^ (MMRC #037156-JAX) line demonstrates reduced fentanyl seeking, despite similar acquisition and fentanyl intake as their wildtype littermates. Drd1-cre^120Mxu^ mice also show reduced fentanyl conditioned place preference despite increased fentanyl-induced hyperlocomotion, as well as increased sensitivity to D1 receptor agonism, but normal sucrose self-administration and seeking. We further show these mice have differential mRNA expression of several NAc MSN markers both at baseline and after prolonged abstinence from fentanyl self-administration, including elevated *Drd1* expression at both timepoints. Lastly, we show that chemogenetic stimulation of ventral mesencephalon-projecting NAc core MSNs (putative D1-MSNs) in wildtype mice recapitulates the blunted fentanyl seeking exhibited by Drd1-cre^120Mxu^ mice, supporting aberrant signaling in NAc D1-MSNs as a mechanism promoting this behavior in the Drd1-cre^120Mxu^ line.

## Materials and methods

### Experimental subjects

All procedures were approved by the Institutional Animal Care and Use Committee at the Pennsylvania State University College of Medicine (PSUCOM) and conducted in accordance with NIH guidelines for the use of laboratory animals. Drd1-cre^120Mxu^ mice on a C57BL/6 J background were obtained from The Jackson Laboratory (B6;129-Tg(Drd1-cre)120Mxu/Mmjax; Jax strain #024860; RRID:MMRRC_037156-JAX) [55]. At PSUCOM, Drd1-cre^120Mxu^ hemizygotes were bred with C57BL/6 J wildtype mice obtained from The Jackson Laboratory. Mice were given food and water *ad libitum* and housed in the PSUCOM vivarium on a 12:12 h light:dark cycle with lights on at 7:00. All mice were housed in corncob bedding and provided with nestlets. Mice were group housed until intravenous surgery, after which they were pair-housed across a perforated acrylic divider throughout self-administration to prevent isolation stress. All experiments used mice of both sexes aged 8–10 weeks at the start of behavior.

### Procedures

All procedures are detailed in Supplemental.

### Statistics

Data were analyzed with Graphpad Prism 10 (La Jolla, CA) and JASP [71]. In the absence of significant sex effects or interactions, we collapsed data by sex. Any repeated measures data (e.g. self-administration, locomotion) were analyzed with repeated-measures ANOVA (RM-ANOVA) employing a Greenhouse-Geisser sphericity correction, with sex and genotype as between-subjects factors. Seeking data were analyzed with ANOVA using sex and genotype as between-subjects factors, and nose-poke as within-subject factor. Data without repeated measures (e.g. conditioned place preference, stereotypy) were analyzed by unpaired *t*-test due to no sex effects, or ANOVA when sex was significant. Gene expression data were analyzed with 2-way ANOVA using sex and genotype. For chemogenetic experiments, seeking was analyzed with ANOVA using sex, genotype, and virus as between factors, and nose-poke as within-subject factor. c-fos expression was analyzed with Welch’s ANOVA due to unequal variance. All post-hoc tests employed Sidak’s correction, except for the chemogenetic conditioned place preference and c-fos experiments which employed Dunnett’s comparing to the mCherry control.

## Results

### Drd1-cre^120Mxu^ mice exhibit attenuated fentanyl seeking behavior

**Fig. 1:**
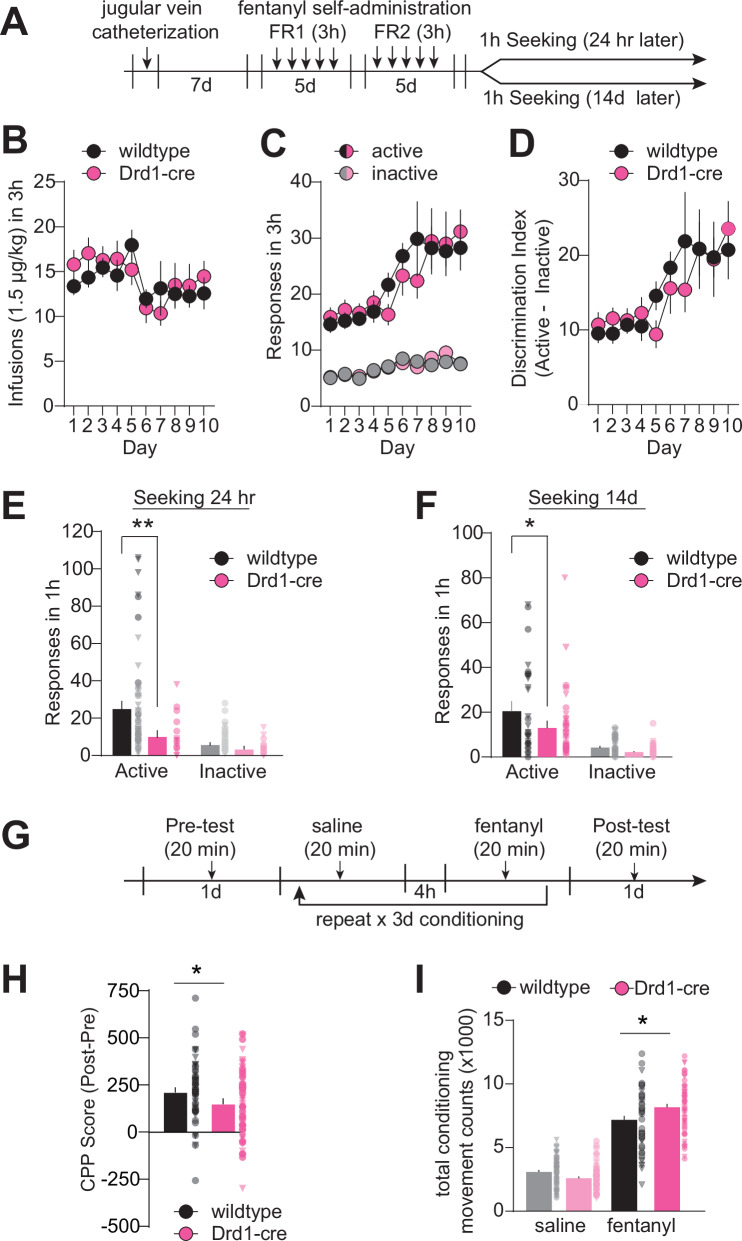
Drd1-cre^120Mxu^ mice show blunted fentanyl seeking relative to wildtype mice. **A** Experimental timeline for self-administration. Following recovery from jugular vein catheter surgery, mice underwent 5 days of fentanyl self-administration training (3 h sessions) under fixed ratio 1 (FR1), followed by 5 days under FR2. Half of the mice underwent a non-reinforced seeking test 24 h after the last self-administration session and the other half underwent seeking 14 d later. **B** Number of fentanyl infusions (1.5 µg/kg) earned during self-administration training in Drd1-cre^120Mxu^ (magenta) and wildtype (black) mice during training (wildtype *n* = 38♀, 48♂; Drd1-cre^120Mxu^ *n* = 27♀, 26♂). **C** Number of active and inactive responses during fentanyl self-administration training. **D** Discrimination index (active minus inactive responses) during fentanyl self-administration training. **E** Number of active and inactive responses during a 1h non-reinforced seeking test in wildtype and Drd1-cre^120Mxu^ mice (**, *p* = 0.026. Sidak’s post-hoc after 2-way ANOVA. Triangles denote datapoints from males and circles from females). **F** Number of active and inactive responses during a seeking test in separate mice after 14 days abstinence (*, *p* = 0.03, Sidak’s post-hoc after 2-way ANOVA). **G** Experimental timeline for conditioned place preference. Mice freely explored the apparatus for 20 min during the pre-test day. For the following three days, mice received saline (10 mL/kg ip) in one compartment, followed 4 h later by fentanyl (0.2 mg/kg ip) in the other. On the fifth day, mice freely explored the apparatus. **H** Preference for the fentanyl-paired compartment expressed as time spent in fentanyl-paired chamber during the post-test minus time spent during pre-test (*, *p* = 0.03, unpaired *t*-test. Wildtype *n* = 33♀, 30♂; Drd1- cre^120Mxu^ *n* = 33♀, 32♂). **I** Total number of movement counts during conditioning sessions (*, *p* = 0.0047, Sidak’s post-hoc after 2-way ANOVA). Data are presented as mean ± SEM with individual mice overlaid.

NAc dopamine D1 receptors (D1R) play key roles in mediating reward and addiction phenotypes [9]. To answer questions about the mechanisms of D1-neurons in fentanyl use and relapse, we obtained Drd1-cre^120Mxu^ [55] mice from the Jackson Laboratory. While this Drd1-cre line has been used by other investigators in recent papers [56–70], known issues in other lines [72–75] necessitate characterizing behavior of all transgenic mice. Thus, we first asked if Drd1-cre^120Mxu^ mice would self-administer intravenous fentanyl. Similar to our previous work in wildtype C57/BL6J mice [76], Drd1-cre positive and negative mice underwent 10 d of fentanyl self-administration training (timeline in Fig. 1A). Both genotypes self-administered a similar number of fentanyl infusions (Fig. 1B, sexes combined due to no sex effects or interactions. RM-ANOVA, Day: F_3.030,415.2_ = 3.40, *p* = 0.018; Day x Genotype: F_9,1233_ = 1.06, *p* = 0.39). Both genotypes learned to discriminate between active and inactive nose-pokes as measured by discrimination index (Fig. 1C, D, RM-ANOVA, Day: F_2.975, 407.6_ = 6.27, *p* = 0.0004; Day x Genotype: F_9,1233_ = 0.67, *p* = 0.72). Across 10 d of self-administration, both genotypes had similar total fentanyl intake (wildtype female: 0.24 ± 0.02, male: 0.19 ± 0.02; Drd1-cre female: 0.21 ± 0.02, male: 0.22 ± 0.04 mg/kg).

We next asked if Drd1-cre^120Mxu^ mice exhibited fentanyl seeking under extinction conditions. As expected, after 24 h abstinence, wildtype mice exhibited fentanyl seeking behavior marked by increased responding at the active nose-poke. By contrast, Drd1-cre^120Mxu^ mice exhibited reduced fentanyl seeking behavior (Fig. 1E, 2-way ANOVA, Genotype: F_1,71_ = 5.2, *p* = 0.02; Active nose-poke, wildtype vs. Drd1-cre, *p* = 0.006, Sidak’s). We tested a separate set of mice after 14 d abstinence. Like the 24 h timepoint, Drd1-cre^120Mxu^ mice exhibited reduced fentanyl seeking behavior relative to wildtype mice (Fig. 1F, 2-way ANOVA, Genotype: F_1,64_ = 4.23, *p* = 0.04; Active nose-poke, wildtype vs. Drd1-cre^120Mxu^, *p* = 0.03, Sidak’s). To see if this generalized to other forms of fentanyl reward, we used conditioned place preference (CPP) in a separate set of mice (Timeline in Fig. 1G). We found slightly reduced CPP scores in Drd1-cre^120Mxu^ mice relative to wildtype (Fig. 1H, t_1,126_ = 2.17, *p* = 0.03). When we examined fentanyl-induced hyperlocomotion, another proxy for drug reward, we found Drd1-Cre^120Mxu^ mice exhibit *greater* movement counts during fentanyl conditioning (Fig. 1I, drug x genotype: F_1,109_ = 13.22 *p* = 0.0004; wildtype vs. Drd1-cre^120Mxu^ fentanyl, *p* = 0.0047, Sidak’s). To determine if reduced drug reward was restricted to fentanyl, we asked if this generalized to other drugs with misuse potential, and compared cocaine self-administration and seeking in Drd1-cre^120Mxu^ mice relative to wildtype mice in our published dataset [77] (Supplemental Fig. 1A). Unlike fentanyl, Drd1-cre^120Mxu^ had reduced cocaine intake relative to wildtype mice (Supplemental Fig. 1B, C, Infusions: Genotype: F_1,24_ = 10.8, *p* = 0.003; Active responses: Genotype F_1,24_ = 17.3, *p* = 0.0003). Like fentanyl, Drd1-cre^120Mxu^ had fewer active responses during the non-reinforced seeking test at 24 h (Supplemental Fig. 1D, Genotype: F_1,48_ = 22.6, *p* < 0.0001; Active responses, wildtype vs. Drd1-cre^120Mxu^, *p* = 0.0001, Sidak’s). Finally, to determine if Drd1-cre^120Mxu^ mice had generally impaired reward seeking or if this behavior was specific to misused drugs, we compared sucrose self-administration and seeking in Drd1-cre^120Mxu^ mice to wildtype mice (Supplemental Fig. 2A). We found that Drd1-cre^120Mxu^ mice had no difference in number of sucrose pellets earned or consumed (Supplemental Fig. 2B, RMANOVA, Genotype F_1,22_ = 0.2; 2 C F_1,22_ = 0.8, p’s>0.3) and no differences in nose-poke discrimination (Supplemental Fig. 2D, E, Genotype F_1,22_ = 0.2, *p* = 0.7) relative to wildtype littermates. D1-cre^120Mxu^ mice also exhibited similar sucrose seeking as wildtype littermates under both food-restriction and *ad libitum* conditions (Supplemental Fig. 2F, G, Genotype F’s< 0.21, p’s>0.6).

### Drd1-cre^120Mxu^ mice have altered baseline gene expression in nucleus accumbens

**Fig. 2:**
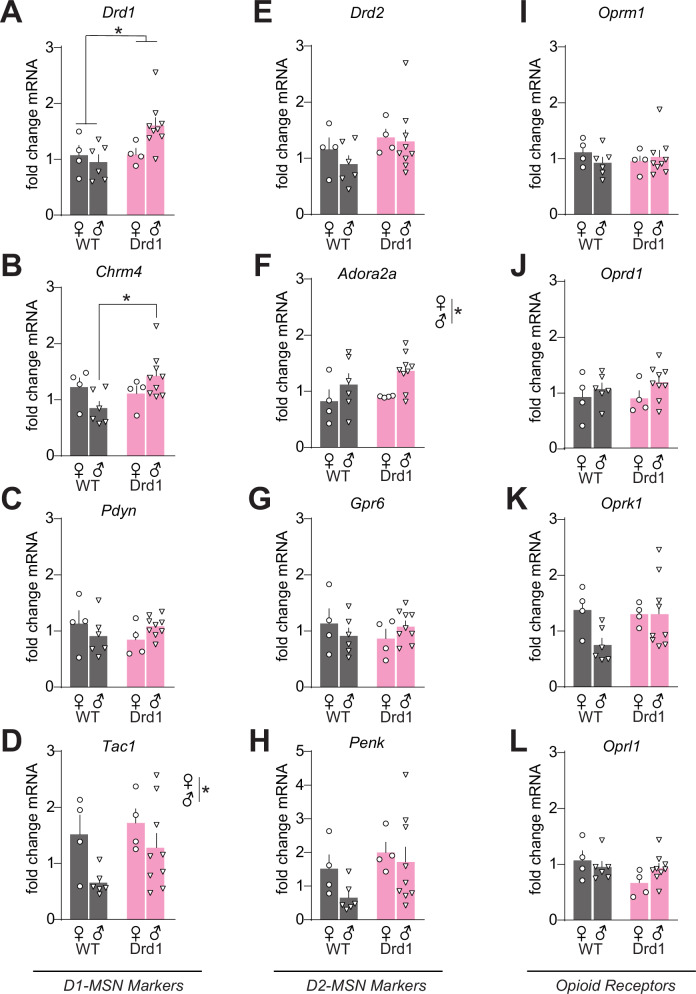
Drd1-cre^120Mxu^ mice have elevated expression of medium spiny neuron marker genes in nucleus accumbens at baseline. Fold change mRNA in experimentally naïve wildtype (black) and Drd1-cre^120Mxu^ (magenta) mice, relative to average of naïve male and female wildtype mice (wildtype *n* = 4♀, 6♂; Drd1-cre^120Mxu^ *n* = 4♀, 9♂). **A** Dopamine D1 receptor, *, *p* = 0.045, main effect of genotype. **B** Muscarinic acetylcholine receptor M4, Sex x Genotype Interaction, *p* = 0.036; * *p* = 0.01, wildtype vs. Drd1-cre males, Sidak’s post-hoc. **C** Preprodynorphin. **D** Preprotachykinin-1, *, *p* = 0.026, main effect of sex. **E** Dopamine D2 receptor. **F** Adenosine A2a receptor, *, *p* = 0.028, main effect of sex. **G** G protein-coupled receptor 6. **H** Preproenkephalin. **I** Mu opioid receptor. **J** Delta opioid receptor. **K** Kappa opioid receptor. **L** Opioid related nociceptin receptor 1. Data are mean ± SEM with individual mice overlayed.

Previous work using Drd2-EGFP mice found elevated striatal *Drd2* expression with effects on behavioral responses to cocaine [72]. Thus, we next asked if Drd1-cre^120Mxu^ mice also have altered baseline expression of dopamine receptors that might drive the behavioral phenotypes. Given the importance of NAc in opioid-reward and opioid seeking [1], we looked at gene expression in total NAc. In experimentally naïve mice, we found elevated *Drd1* expression in both sexes of Drd1-cre^120Mxu^ mice compared to wildtype littermates (Fig. 2A, 2-way ANOVA, Genotype: F_1,19_ = 4.60, *p* = 0.045). Increased *Drd1* mRNA is not due to an increase in the number of D1 relative to D2 MSNs, as viral labeling with a Cre-switch virus [78] revealed typical balance in D1 (Cre positive) vs. D2 (Cre negative) cells in NAc. (52.6 ± 4.4% D1, 47.4 ± 4.4% D2; Supplemental Fig. 3A–C). We also found elevated expression of D1-MSN marker *Chrm4* in males (Fig. 2B, Sex x Genotype: F_1,19_ = 5.07, *p* = 0.036; wildtype vs. Drd1-cre male *p* = 0.01, Sidak’s). Not all D1-MSN markers were elevated, as there were no differences in *Pdyn* (Fig. 2C), and only baseline sex differences in *Tac1* (Fig. 2D, Sex: F_1,19_ = 5.85, *p* = 0.026). We found no changes in D2-MSN markers *Drd2*, *Adora2a, Gpr6*, or *Penk* (Fig. 2E–H) in Drd1-cre^120Mxu^ mice, with only a baseline sex difference in *Adora2a* (Fig. 2F, Sex: F_1,19_ = 5.67, *p* = 0.028). These differences in MSN marker genes did not generalize to all Drd1-cre mice, as we found no aberrant expression in a different Drd1-cre line (GensatFK150; Supplemental Fig. 3D). Given the differences in fentanyl seeking between genotypes, we also looked at expression of opioid receptors and related nociceptin receptor in NAc. In experimentally naïve mice, we found no differences in opioid receptor expression between genotypes (Fig. 2I–L).

### Drd1-cre^120Mxu^ mice exhibit increased sensitivity to D1 receptor activation

**Fig. 3:**
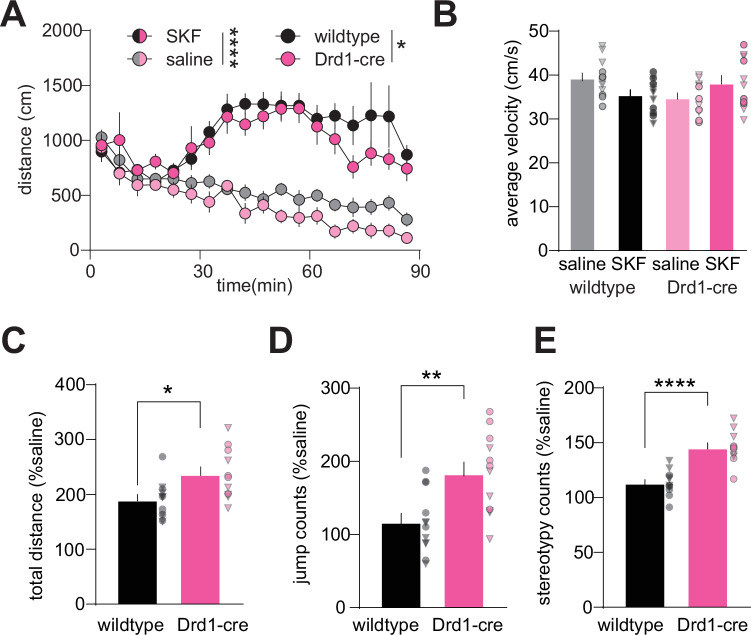
Drd1-cre^120Mxu^ mice have attenuated locomotor response to a novel environment and show increased sensitivity to a D1 receptor agonist. Male and female wildtype (black) and Drd1-cre^120Mxu^ (magenta) mice received saline or 30 mg/kg SKF-38393 s.c. prior to open field testing (wildtype saline *n* = 6♀, 6♂; wildtype SKF *n* = 6♀, 6♂; Drd1-cre saline *n* = 5♀, 6♂; Drd1-cre SKF *n* = 5♀, 6♂). **A** Distance traveled in the open field over 90 min in 5 min bins. ****, *p* < 0.0001, main effect of drug; *, *p* = 0.044, main effect of genotype. **B** Average velocity in the open field in saline and SKF treated mice. Triangles represent data points from male mice and circles from females. **C** Total distance traveled in SKF-treated wildtype and Drd1-cre relative to saline-treated mice. *, *p* = 0.011, unpaired *t*-test. **D** Jump counts in SKF-treated mice relative to saline-treated mice. **, *p* = 0.003, unpaired *t*-test. **E** Stereotypy counts in SKF-treated mice relative to saline-treated mice. ****, *p* < 0.0001, unpaired *t*-test. Data are presented as mean ± SEM with individual mice overlaid.

Since Drd1-cre^120Mxu^ mice had elevated *Drd1* expression in NAc, we next asked if this would produce a baseline locomotor phenotype or increased sensitivity to D1R agonists. We administered D1R agonist SKF-38393 (SKF, 30 mg/kg sc), or saline, just prior to open field testing, which typically produces hyperlocomotion in unhabituated mice [79]. Both SKF-treated wildtype and Drd1-cre^120Mxu^ mice exhibited increased locomotion relative to their saline-treated counterparts (Fig. 3A, time x drug: F_4.9,186.4_= 7.39, *p* < 0.001). Contrary to our prediction of increased distance traveled, both saline- and SKF-treated Drd1-cre^120Mxu^ traveled less total distance across the 90 min relative to wildtype (Genotype: F_1,38_ = 4.1, *p* = 0.049), that was not mediated by differences in average velocity (Fig. 3B). Given the baseline differences in locomotion, we next looked at distance traveled as a percent of saline treatment. Drd1-cre^120Mxu^ mice showed greater SKF-potentiated locomotion relative to wildtype mice, suggesting increased D1R sensitivity (Fig. 3C, t_1,21_ = 2.79, *p* = 0.011). Since D1R activation can elicit stereotyped movements [80], we also looked at jumps and stationary movement counts (stereotypy counts). Drd1-cre^120Mxu^ mice exhibited more SKF-induced jumps (Fig. 3D, t_1,21_ = 3.33, *p* = 0.003) and stereotypy counts (Fig. 3E, t_1,21_ = 5.9 *p* < 0.0001). Together, these data suggest Drd1-cre^120Mxu^ mice have reduced locomotion in a novel environment, but increased sensitivity to D1R activation.

### Drd1-cre^120Mxu^ mice exhibit dysregulated nucleus accumbens gene expression after fentanyl experience

**Fig. 4:**
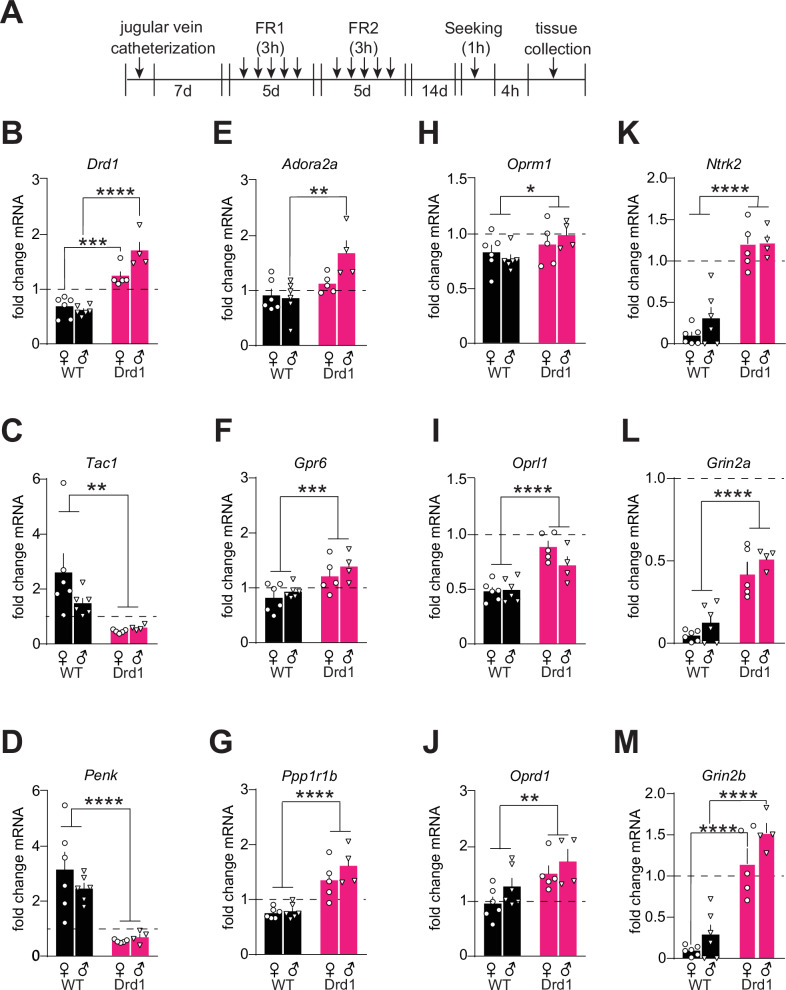
Molecular adaptations in nucleus accumbens differ across genotype following abstinence from fentanyl self-administration. **A** Experimental timeline. Following recovery from jugular vein catheter surgery, mice underwent 5 days of fentanyl self-administration under fixed ratio 1 (FR1), followed by 5 days under FR2. Following 14 days of homecage abstinence, mice underwent a non-reinforced seeking test, then nucleus accumbens tissue was collected ~4 h later. Fold change mRNA relative to experimentally naïve male and female wildtype mice (dashed line; wildtype *n* = 6♀, 6♂; Drd1-cre^120Mxu^ n = 5♀, 4♂). **B** Dopamine D1 receptor, Sex x Drug Interaction, *p* = 0.0087; ***p = 0.0005, ****p < 0.0001, wildtype vs. Drd1-cre females, males, respectively, Sidak’s post-hoc. **C** Preprotachykinin-1, **p = 0.0021, main effect of genotype. **D** Preproenkephalin, ****p < 0.0001, main effect of genotype. **E** Adenosine A2a, Sex x Drug Interaction, *p* = 0.044; **p = 0.0018, wildtype vs. Drd1-cre males, Sidak’s post-hoc. **F** G protein-coupled receptor 6, ***p = 0.001, main effect of genotype. **G** Dopamine and cAMP-regulated phosphoprotein (DARPP-32), ****p < 0.0001, main effect of genotype. **H** Mu opioid receptor, *p = 0.041, main effect of genotype. **I** Opioid related nociceptin receptor 1, ****p < 0.0001, main effect of genotype. **J** Delta opioid receptor, **p = 0.005, main effect of genotype. **K** Tropomyosin receptor kinase B (TrkB) receptor, **** *p* < 0.0001, main effect of genotype. **L** NMDA receptor Subunit 2 A, ****p < 0.0001, main effect of genotype. **M** NMDAR Subunit 2B, ****p’s<0.0001, wildtype vs. Drd1-cre females, males, respectively, Sidak’s post-hoc. Data are presented as mean ± SEM with individual mice overlaid.

Given the increase in *Drd1* expression, and increased response to a D1R agonist in experimentally naïve Drd1-cre^120Mxu^ mice, we next asked if elevated *Drd1* expression was maintained in mice with fentanyl self-administration experience, as this may underlie their behavioral differences (Timeline in Fig. 4A, behavior in Supplemental Fig. 4B). After 14 d abstinence, fentanyl-experienced Drd1-cre^120Mxu^ mice maintained elevated *Drd1* expression relative to naïve wildtype mice (Drd1-cre females: 1.25 ± 0.09, males: 1.7 ± 0.16; Fig. 4B, Sex x Genotype: F_1,17_ = 8.78, *p* = 0.009; females: *p* = 0.0005, males: *p* < 0.0001, Sidak’s). This is juxtaposed against a relative *Drd1* downregulation in fentanyl-experienced wildtype mice (wildtype females: 0.69 ± 0.08, males: 0.62 ± 0.04; Fig. 4B). We also looked beyond *Drd1*, and additional genotype differences emerged that were not present in naïve mice. D1-MSN marker *Tac1* was downregulated in Drd1-cre^120Mxu^ mice with fentanyl experience (females: 0.47 ± 0.029, males: 0.6 ± 0.051; Fig. 4C, Genotype: F_1,17_ = 13.10, *p* = 0.002). D2-MSN marker *Penk* was also downregulated in fentanyl-experienced Drd1-cre^120Mxu^ mice (females: 0.54 ± 0.02, males: 0.68 ± 0.12), juxtaposed against a relative upregulation in fentanyl-experienced wildtype mice (female: 3.15 ± 0.06; male: 2.46 ± 0.20; Fig. 4D, Genotype: F_1,17_ = 30.7, *p* < 0.0001). Additional D2-MSN marker genes *Adora2a* and *Gpr6* were now upregulated in fentanyl-experienced Drd1-cre^120Mxu^ mice, although *Adora2a* was only significantly upregulated in males (Fig. 4E, F, *Adora2a*, Sex x Genotype: F_1,17_ = 4.7, *p* = 0.044, male wildtype vs. Drd1-cre, *p* = 0.0018; *Gpr6*, Genotype: F_1,17_ = 15.8, *p* = 0.001). We also found upregulation of general MSN marker *Ppp1r1b* (DARPP-32) in fentanyl-experienced Drd1-cre^120Mxu^ mice (Fig. 4G, Genotype F_1,17_ = 43.5, *p* < 0.0001), a difference that was absent in experimentally naïve mice (Supplemental Fig. 5A). When we looked at opioid receptor expression, we found differences that were absent in experimentally naïve mice. Compared to fentanyl-experienced wildtype mice, Drd1-cre^120Mxu^ mice had less downregulation of *Oprm1* (wildtype females: 0.82 ± 0.07, males: 0.76 ± 0.04; Drd1-cre females: 0.90 ± 0.09, males 0.98 ± 0.06; Fig. 4H, Genotype: F_1,17_ = 4.9, *p* = 0.04) and *Oprl1* (wildtype females: 0.48 ± 0.04, males: 0.49 ± 0.04; Drd1-cre females: 0.88 ± 0.06, males: 0.71 ± 0.08; Fig. 4I, Genotype: F_1,17_ = 36.7, *p* < 0.0001). Drd1-cre^120Mxu^, but not wildtype mice, also upregulated *Oprd1* (wildtype females: 0.95 ± 0.12, males: 1.26 ± 0.15; Drd1-cre females: 1.49 ± 0.15, males: 1.72 ± 0.22; Fig. 4J, Genotype F_1,17_ = 10.2, *p* = 0.0005). Because chronic opioid exposure is associated with reduction in NAc TrkB expression [17] and NAc NMDAR function [81], we also looked at expression of *Ntrk2*, and GluN2 subunits *Grin2a* and *Grin2b*. In fentanyl-experienced wildtype mice, we found expected downregulation of *Ntrk2* that was completely absent in Drd1-cre^120Mxu^ mice (wildtype female: 0.10 ± 0.044, male: 0.31 ± 0.013; Drd1-cre female: 1.17 ± 0.12, male: 1.23 ± 0.09; Fig. 4K, Genotype: F_1,17_ = 88.25, *p* < 0.0001). Similarly, downregulation of GluN2 subunits was more extensive in fentanyl-experienced wildtype mice compared with Drd1-cre^120Mxu^, especially for *Grin2b* (*Grin2a*: wildtype female 0.05 ± 0.01, male 0.12 ± 0.05; Drd1-cre female 0.43 ± 0.07, male 0.51 ± 0.03, Genotype F_1,17_ = 76.1, *p* < 0.0001; *Grin2b*: wildtype female 0.09 ± 0.02, male 0.29 ± 0.012; Drd1-cre female 1.15 ± 0.18, male 1.5 ± 0.11, Genotype F_1,17_ = 91.0, *p* < 0.0001; Fig. 4L, M) None of these differences were present in experimentally naïve mice (Supplemental Fig. 5B–D), nor did they arise due to different fentanyl exposure (qPCR subset, total intake Drd1-cre 0.21 ± 0.07, wildtype 0.25 ± 0.08 mg/kg).

### Chemogenetic activation of putative D1-MSNs in wildtype mice suppresses fentanyl seeking

**Fig. 5:**
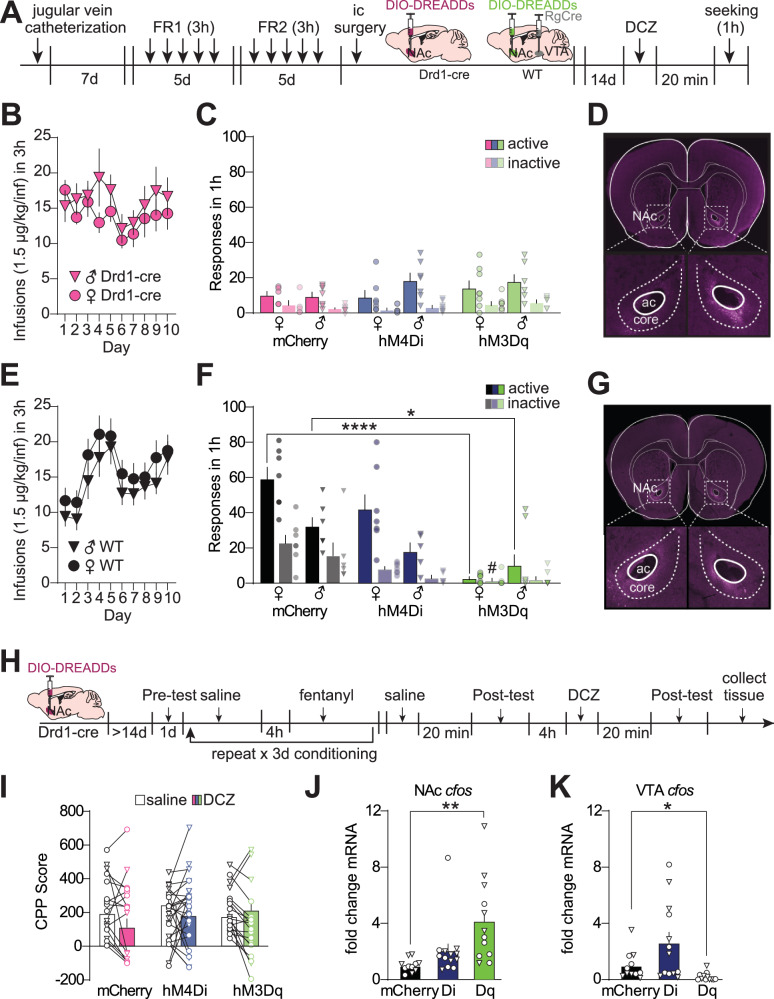
Chemogenetic manipulation of NAc D1-MSNs blunts fentanyl seeking in wildtype mice but does not alter seeking in Drd1-cre^120Mxu^ mice. **A** Experimental timeline for IVSA experiments. Following jugular vein catheter surgery mice underwent 10 days of fentanyl self-administration training. Mice then underwent surgery to express DREADDs in NAc core D1-MSNs, using Cre-dependent DREADDs in NAc core of Drd1-cre^120Mxu^ mice, or retrograde Cre in ventral mesencephalon and Cre-dependent DREADDs in NAc core of wildtype mice. Following 14 d abstinence and viral expression, mice were given 0.1 mg/kg DCZ i.p. 20 min prior to a 1h seeking test. **B** Infusions earned during fentanyl self-administration in male and female Drd1-cre^120Mxu^ mice. **C** Active and inactive responses during the fentanyl seeking test in Drd1-cre^120Mxu^ mice expressing mCherry control (*n* = 5♀, 7♂), inhibitory hM4Di (*n* = 7♀, 7♂), or stimulatory hM3Dq (*n* = 8♀, 6♂) in NAc core D1-MSNs. **D** Image demonstrating DREADDs expression in Drd1-cre^120Mxu^ mice is restricted to the NAc core (ac, anterior commissure). **E** Fentanyl infusions earned during self-administration in male and female wildtype mice. **F** Active and inactive responses during the fentanyl seeking test in wildtype mice (mCherry, *n* = 7♀, 6♂; hM4Di, *n* = 7♀, 5♂; hM3Dq, *n* = 7♀, 8♂). Active responses: ****p < 0.0001, hM3Dq vs. mCherry females; *p = 0.024, hM3Dq vs. mCherry males, Sidak’s. Inactive responses: #*p* = 0.024, female hM3Dq vs. mCherry. **G** Image demonstrating DREADDs expression in wildtype mice is restricted to the NAc core **H** Experimental timeline for CPP experiments. Drd1-cre^120Mxu^ mice underwent surgery for Cre-dependent DREADDs in NAc. Mice freely explored the apparatus during the pre-test day. For the following three days, mice received saline (10 mL/kg i.p.) in one compartment, followed 4 h later by fentanyl (0.2 mg/kg i.p.) in the other. On the fifth day, mice received saline (or DCZ) 20 min prior to the first post-test. Then, 4 h later, mice received 0.1 mg/kg DCZ (or saline) 20 min prior to the second post-test. Tissue was collected immediately following the second post-test to capture immediate early gene expression. **I** CPP score during the post-test under saline and DCZ conditions (mCherry, *n* = 12♀, 13♂; hM4Di, *n* = 12♀, 7♂; hM3Dq, *n* = 10♀, 6♂). **J** In mice receiving DCZ just prior to tissue collection, immediate early gene *cfos* is upregulated in NAc of Drd1-cre^120Mxu^ mice expressing hM3Dq relative to mCherry, **p = 0.007, Dunnet’s T3 (mCherry, *n* = 5♀, 4♂; hM4Di, *n* = 7♀, 6♂; hM3Dq, *n* = 8♀, 4♂). **K** In downstream VTA, *cfos* is downregulated in hM3Dq relative to mCherry, *p = 0.049, Dunnet’s T3. Data are presented as mean ± SEM with individual mice overlaid.

Due to persistent upregulation of NAc *Drd1* in the Drd1-cre^120Mxu^ mice, we next asked if chemogenetically manipulating activity of NAc D1-MSNs using Designer Receptors Exclusively Activated by Designer Drugs (DREADDs) could restore fentanyl seeking to that of wildtype mice (Timeline in Fig. 5A). Immediately following fentanyl self-administration (Fig. 5B), we assigned Drd1-cre^120Mxu^ mice to groups to ensure similar intake across sex and virus (future virus F_2,33_ = 0.4, future virus x sex F_1,33_ = 0.3). After two weeks of abstinence, we inhibited or activated NAc core D1-MSNs with the DREADDs ligand deschloroclozapine (DCZ) prior to a fentanyl seeking test. We found neither chemogenetic inhibition, nor activation, influenced fentanyl seeking behavior in Drd1-cre^120Mxu^ mice (Fig. 5C, nose-poke x virus F_2,35_ = 2.15, *p* = 0.15). By contrast, chemogenetic activation of ventral mesencephalon-projecting NAc core MSNs (putative D1-MSNs, see Discussion) in wildtype mice suppressed fentanyl seeking (Self-administration in Fig. 5E, seeking in Fig. 5F; Nose-poke x sex x virus F_2,34_ = 3.44, *p* = 0.044; Active nose-pokes, hM3Dq vs. mCherry, male *p* = 0.024, female *p* < 0.0001, Sidak’s). This was not due to differences in self-administration behavior, as all mice, regardless of genotype or sex, had comparable fentanyl intake during acquisition (wildtype female 0.26 ± 0.02, male 0.21 ± 0.02, Drd1-cre female 0.21 ± 0.02, male 0.27 ± 0.04 mg/kg, Genotype F_1,76_ = 0.03). As with fentanyl seeking after self-administration, we found Drd1-cre^120Mxu^ mice were also insensitive to chemogenetic manipulation during fentanyl conditioned-place preference. Using a within-subject design (Timeline in Fig. 5H), we found neither inhibiting, nor activating D1-MSNs significantly altered time spent in the fentanyl-paired chamber (Fig. 5I, drug x virus F_2,54_ = 2.8, *p* = 0.07). This was not due to DCZ inefficacy in Drd1-cre^120Mxu^ mice, as stimulating NAc D1-neurons increased *cfos* in NAc (Fig. 5J, Welch’s ANOVA, F_2,16.51_ = 7.8 *p* = 0.0042, mCherry vs. hM3Dq *p* = 0.007, Dunnet’s T3), and decreased *cfos* in downstream ventral tegmental area (VTA) as expected (Fig. 5K, Welch’s ANOVA, F_2,15.86_ = 7.7, mCherry vs. hM3Dq = 0.049, Dunnet’s).

## Discussion

Here we report that the widely-used Drd1-cre^120Mxu^ mouse line [55] exhibits several behavioral and transcriptional differences compared to wildtype mice. We serendipitously discovered that this line has reduced responding in a nonreinforced fentanyl seeking test following either 24 h or 14 days of abstinence, despite normal fentanyl acquisition. This resistance translates to other fentanyl administration paradigms, as Drd1-cre^120Mxu^ mice also have reduced preference for a fentanyl-paired context, despite increased fentanyl-induced hyperlocomotion. This behavior is specific to misused drugs, since these mice also have reduced self-administration and seeking for cocaine, but not sucrose. These mice also exhibit baseline hypolocomotion in a novel environment, as well as relative increased behavioral response to D1R agonism. Although the mechanism underlying fentanyl resistance in these mice is not fully understood, key differences in gene expression in the Drd1-cre^120Mxu^ mice, alongside evidence that stimulating putative D1-MSNs in wildtype mice produces similarly blunted fentanyl seeking, implicates that this phenotype is, at least in part, due to aberrant signaling in D1-MSNs.

### Atypical drug-associated behavior in Drd1-cre^120Mxu^ mice

We showed that Drd1-cre^120Mxu^ mice have reduced self-administration of cocaine but not fentanyl. Our study is not the first to report on a transgenic line with abnormal drug self-administration behavior; for example, ChAT-Cre mice (B6.FVB(Cg)-Tg(Chat-cre)GM60Gsat/Mmucd) have reduced self-administration of intravenous nicotine [73]. However, our findings are unique in that despite normal acquisition of fentanyl self-administration, Drd1-cre^120Mxu^ mice exhibited attenuated nonreinforced fentanyl seeking. This effect is strikingly robust: the blunted fentanyl seeking is observed at both 24 h and 14 days after self-administration, is also reflected in a reduced preference for a fentanyl-paired context, persists in both paradigms despite bidirectional chemogenetic manipulation of D1-MSNs, and it is specific to drugs as there is no impairment of sucrose seeking in these mice.

In wildtype mice, we found chemogenetic stimulation of putative NAc core D1-MSNs (ventral mesencephalon-projecting) attenuated fentanyl seeking. While reminiscent of D1R agonists decreasing cocaine seeking [82], this was also surprising, since research by O’Neal et al. shows activating VTA-projecting NAc neurons exacerbates heroin seeking in rats [83]. This discrepancy may reflect species differences in D1-MSN collaterals to the ventral pallidum (VP). In mice, over 90% of VTA-projecting MSNs send collaterals to VP [84]; in rats, O’Neal et al. report VTA-projecting MSNs do not send collaterals to VP [83]. MSNs make inhibitory synapses on mostly GABAergic neurons in VP [85]. Activating MSN collaterals in VP would increase inhibitory drive on GABAergic VP neurons, and inhibiting VP GABA neurons attenuates remifentanil seeking [86]. Thus, we suspect reduced fentanyl seeking with NAc core D1-MSN activation is explained by inhibition in VP, although this requires further investigation. It is worth noting these mechanisms may not extend to the NAc shell. For example, systemic D1R agonism attenuates cocaine seeking [82], but when delivered directly to the NAc shell, D1R agonists instead promotes cocaine seeking [87]. Since DREADDs expression in our study was limited to NAc core, future work is necessary to elucidate the specific contributions of NAc subregions to fentanyl seeking.

Unlike wildtype mice, we found that chemogenetic manipulation of D1-MSNs did not affect seeking in Drd1-cre^120Mxu^ mice. It is likely D1-MSN stimulation had no effect in Drd1-cre^120Mxu^ mice because their seeking responses are so low at baseline, they cannot be further attenuated (a “floor effect”); additionally, it is perhaps unsurprising that chemogenetic inhibition of D1-MSNs did not increase seeking in Drd1-cre^120Mxu^ mice since this did not increase seeking in wildtype mice. Importantly, the lack of response in Drd1-cre^120Mxu^ mice does not reflect an inability for DREADDs to stimulate or inhibit D1-MSNs, as we identified expected increases and decreases in *cfos* expression after DCZ treatment. However, the DREADDs manipulations in wildtype and Drd1-cre^120Mxu^ are not identical. In Drd1-cre^120Mxu^ mice, we expressed DREADDs in all NAc core D1-MSNs; in wildtype mice, we targeted only ventral mesencephalon-projecting MSNs in NAc core. While these neurons almost exclusively express D1 receptor, there may be minor differences in the populations targeted by both approaches, given the collateralization discussed above.

It is worth noting that neither Drd1-cre^120Mxu^ nor wildtype mice demonstrated increased fentanyl seeking after a longer period of abstinence, a phenomenon termed “incubation of craving” that is well-documented for other misused drugs [88, 89]. A potential explanation is the time course of our seeking experiments. Indeed, incubation of heroin seeking follows an inverted U-shaped time course, with higher responding at 6 and 12 days compared to 1 and 25 [90]. The specific time course for incubation of fentanyl seeking in mice is unknown, although we suspect it arises later than the 14 day timepoint tested here, since rats demonstrate incubation of fentanyl seeking after 30 days [91], and mice exhibit high rates of fentanyl seeking after 4 weeks [92]. Additional work is necessary to fully understand the nuance of time-dependent changes to fentanyl seeking behavior, and whether this differs between genotypes.

### Atypical locomotor responses in Drd1-cre^120Mxu^ mice

We showed that Drd1-cre^120Mxu^ mice exhibit several locomotor phenotypes, including increased locomotor response to fentanyl, reduced locomotion in a novel environment, and increased sensitivity to a D1R agonist. There are several reports on transgenic mouse lines with abnormal locomotor responses to misused drugs. For example, Drd2-EGFP mice [93] have increased striatal *Drd2* expression and reduced locomotor response to cocaine [72]. Similarly, DAT-Ires-Cre mice (B6.SJL-Slc6a3^tm1.1(cre)Bkmn^/J, Jackson Laboratory No: 006660) have reduced dopamine transporter (DAT) expression [94] and function [95], and exhibit attenuated amphetamine-induced locomotion [74] that generalizes to a different DAT-Cre line (Slc6a3^tm1(cre)Xz^, Jackson Laboratory No: 020080) [75]. Here we show Drd1-cre^120Mxu^ mice have elevated expression of NAc *Drd1* and a greater locomotor response to fentanyl. D1-MSNs form inhibitory synapses on GABAergic interneurons in VTA [96, 97]. Increased D1R activation may potentiate dopamine neuron disinhibition, increasing the locomotor response to fentanyl. In support of increased D1R sensitivity, Drd1-cre^120Mxu^ mice exhibited a relatively stronger locomotor stimulatory effect with D1R agonist SKF-38393. Interestingly, Drd1-cre^120Mxu^ mice exhibit baseline hypolocomotion in a novel environment. Altered baseline locomotion is similar to observations in the Drd2-EGFP and the DAT-IRES-Cre line, which exhibit baseline hyperactivity in a novel environment [72, 74] and dysregulated dopamine regulation mechanisms [72, 95]. These baseline locomotor differences are to be expected given the importance of D1- and D2-MSN signaling for normal locomotion [98]. Importantly, the increase in *Drd1* does not reflect an increase in the number of D1-MSNs relative to D2-MSNs in NAc, as our viral labeling indicates there are an equivalent number of D1 positive and negative neurons in Drd1-cre^120Mxu^ mice.

### Nucleus accumbens transcriptional adaptations implicate potential mechanisms of fentanyl resistance

Although the precise mechanisms underlying reduced fentanyl seeking in Drd1-cre^120Mxu^ mice are unknown, looking at the transcriptional differences provides some insight. At baseline, we found Drd1-cre^120Mxu^ mice had greater *Drd1* expression relative to wildtype mice. Increased *Drd1* was maintained following fentanyl self-administration in Drd1-cre^120Mxu^ mice, resulting in an exaggerated genotype difference, since fentanyl-experienced wildtype mice *downregulated Drd1* expression. *Tac1*, another D1-MSN marker, demonstrated baseline sex differences that are consistent with findings in rat striatum [99], but did not initially differ between genotypes. Following fentanyl, wildtype mice upregulated *Tac1*, similar to findings in morphine-exposed rats [51]. In stark contrast, Drd1-cre^120Mxu^ mice downregulated *Tac1* after fentanyl. *Tac1* encodes the precursor for Substance P, a neuropeptide that is induced by morphine [100] and particularly relevant to opioid-dependent behaviors [101]. Substance P is released by local MSN collaterals [102] onto cholinergic interneurons expressing Substance P receptors [103, 104], where it participates in an excitatory microcircuit that promotes coordinated activity between D1- and D2-MSNs [105]. The downregulation of *Tac1* in the NAc of Drd1-cre^120Mxu^ mice likely impairs the function of this local circuit, and as such, may contribute to the blunted fentanyl seeking in these mice.

We also looked at NAc expression of D2-MSN markers, including *Drd2*, *Gpr6*, *Adora2a*, and *Penk*. While expression of these genes did not differ between Drd1-cre^120Mxu^ and wildtype mice at baseline, fentanyl-experienced Drd1-cre^120Mxu^ mice had increased *Gpr6* expression in both sexes, and increased *Adora2a* expression in males. *Gpr6*, which encodes G protein-coupled receptor 6, is enriched in NAc indirect pathway MSNs, where it plays a role in regulating instrumental conditioning [10], but a link to drug misuse has not yet been identified. *Adora2a* encodes adenosine A_2A_ receptors, which are colocalized on D2-MSNs [106, 107] and antagonize activity at the D2 receptor [108]. NAc A_2A_ activity has been shown to regulate cocaine seeking [109], but its role in opioid use is less studied. Two of the most promising molecular targets are *Penk* (encoding the enkephalin precursor) and *Oprd1* (encoding delta opioid receptor, which binds enkephalin). The enkephalinergic system has close interactions with reward pathways: enkephalins released from D2-MSNs modulate neurotransmission in the VP, and enkephalins released locally in NAc modulate NAc signaling at various sites [110]. Prior studies in rats implicated a direct role for NAc *Penk* in opioid seeking, where *Penk* overexpression in NAc potentiates heroin seeking [111], consistent with upregulated *Penk* in our fentanyl-seeking wildtype mice. Since Drd1-cre^120Mxu^ mice downregulate *Penk*, and upregulate *Oprd1* only after fentanyl, these adaptations could drive decreased fentanyl seeking and warrant further investigation.

In addition to the MSN markers, we also investigated other genes that, in the NAc, have known roles in opioid misuse. We found that *Ntrk2*, which encodes BDNF receptor TrkB, was downregulated following fentanyl abstinence in wildtype mice but was unchanged in Drd1-cre^120Mxu^ mice. This finding is consistent with previous work showing morphine exposure downregulates TrkB in D1-MSNs, resulting in impaired BDNF signaling that promotes morphine reward [17]. It is tempting to speculate that, given unchanged *Ntrk2* expression, Drd1-cre^120Mxu^ mice preserve normal BDNF signaling in D1-MSNs after fentanyl, thereby reducing fentanyl seeking. We also found Drd1-cre^120Mxu^ mice have blunted downregulation of *Grin2a* and *Grin2b* following fentanyl abstinence. These genes encode the GluN2 subunits of the glutamatergic NMDA receptor. NAc glutamatergic signaling is essential for opioid seeking [112], and previous work shows chronic opioid use alters NMDA-mediated signaling in NAc [81]. The observed downregulation of NMDA receptor components in wildtype mice aligns with findings of an increased NAc AMPA/NMDA ratio that results from chronic morphine use and persists throughout protracted withdrawal [32, 35]. This adaptation is believed to contribute to an increased excitatory drive onto D1-MSNs that promotes drug seeking [113]. A similar adaptation results from fentanyl abstinence [52], and this process is likely disrupted in Drd1-cre^120Mxu^ mice, thereby impairing fentanyl seeking.

## Conclusions

Although we did not identify the precise circuit and molecular mechanisms underlying reduced relapse to fentanyl seeking in Drd1-cre^120Mxu^ mice, it is clear these mice have aberrant NAc D1-MSN adaptations with implications for circuit function. The fentanyl abstinence-induced changes in NAc gene expression we observed in Drd1-cre^120Mxu^ mice suggest some of these adaptations include impaired Substance P-dependent and enkephalinergic signaling, conserved BDNF-dependent signaling, and altered NMDA plasticity, all of which could reduce fentanyl seeking, although additional experiments are necessary to confirm these possibilities, including fully characterizing cell-type and subregion differences in gene expression. A recent study proposed that imbalanced D1- to D2-MSN plasticity promotes negative affect during opioid abstinence, and restoring this plasticity through a D1R-dependent process attenuated negative affect and relapse [114]. Given that Drd1-cre^120Mxu^ mice exhibit increased sensitivity to D1R activation, combined with the potential adaptations to NAc plasticity, it is tempting to speculate that the Drd1-cre^120Mxu^ mice preserve D1- to D2-MSN balance after fentanyl experience. Future work should investigate whether Drd1-cre^120Mxu^ mice exhibit abstinence-induced negative affective behavior comparable to wildtype mice, as the neuroadaptations described here may promote resilience during abstinence, thereby decreasing relapse. In conclusion, our findings provide potential new molecular mechanisms and neurobiological targets for understanding susceptibility and resistance to fentanyl relapse.

## Supplementary information

Supplemental Material

## Data Availability

All gene expression, and the majority of behavioral data are presented as individual data points in either main or supplemental figures. Any additional behavioral data are available from the corresponding author upon reasonable request.
