# Supplementary material for: A Drd1-cre mouse line with nucleus accumbens gene dysregulation exhibits blunted fentanyl seeking
Source: Neuropsychopharmacology. 2025 May 2;50(13):1993–2005. doi: 10.1038/s41386-025-02116-0 (PMC12603089; doi:10.1038/s41386-025-02116-0)
Supplement: Supplementary file 1 — Supplemental Material [file 41386_2025_2116_MOESM1_ESM.docx]

**Supplemental Methods**

*Drugs*

Fentanyl citrate (#22659), cocaine hydrochloride (#22165), and SKF-38393 hydrobromide (#19150), were obtained from Cayman chemical (Ann Arbor, MI). Deschloroclozapine (#7193) was obtained from Tocris Bioscience (Minneapolis, MN). All other veterinary agents (e.g. ketamine, xylazine, enrofloxacin, etc.) were obtained from PSUCOM Animal Resources. All drugs were dissolved in sterile saline (0.9%).

*Intravenous catheter surgery*

At 7-8 weeks of age, mice were implanted with indwelling jugular vein catheters (One Channel Vascular Access Button™ for Mice, Instech) under ketamine-xylazine anesthesia using previously described procedures [76]. Mice were allowed 7d to recover from surgery, during which they received daily subcutaneous injections of antibiotics (enrofloxacin 20 mg/kg) and Lactated Ringer’s solution (10 mL/kg). Implanted catheters were flushed daily with 30 μL of heparinized saline containing enrofloxacin (200 IU/mL heparin, 0.227% enrofloxacin).

*Fentanyl self-administration and seeking*

Mice underwent intravenous fentanyl self-administration as previously described [76]. Briefly, mice were habituated to the operant chambers (MED Associates, Saint Albans, VT, USA) for 30 minutes one day prior to starting self-administration. Mice then underwent 10 consecutive days of fentanyl self-administration, the first 5d under a fixed-ratio 1 (FR1) schedule of reinforcement, followed by 5d under FR2 (3 h/day, 1.5 μg/kg/infusion). Sufficient responses in the active nose-poke triggered a 10 μL fentanyl infusion, turned off the house-light and the active nose-poke light, and illuminated the cue light above the active nose-poke for 1s. Any additional active responses during the 1s infusion period were recorded but did not result in further infusions (‘timeout’). Responses on the inactive nose-poke were recorded but were without programmed consequences. No prior training was used for FR1 acquisition. After self-administration, all mice underwent a 1h seeking test under extinction conditions in which responses in the active nose-poke resulted in cue presentations but no drug delivery. Mice underwent the seeking test either 24h or 14d after the last self-administration session. To better match sample sizes, we added additional wildtype mice that were bred in our colony over the same time period, with parents also purchased from Jax (P’s>0.8 for Drd1-cre^120Mxu^ wildtype vs non- Drd1-cre^120Mxu^ wildtype, ~50% littermates from Drd1-cre^120Mxu^ ). Tissue was collected approximately 4 hours after the last seeking test, and all gene expression data compared Drd1-cre^120Mxu^ positive mice to their wildtype littermates.

*Fentanyl conditioned place preference*

Conditioned place preference (CPP) was performed in a three-chambered rectangular apparatus consisting of a neutral center compartment and two side compartments with different contextual cues, connected by automatic guillotine doors, and equipped with infrared photobeam detectors that track animal position and movement (ENV-3013, MED Associates). On the first day of testing (pre-test), mice underwent a 5 min habituation in the center compartment, after which the doors opened, and they freely explored the entire apparatus for 20 min. The least-preferred chamber was subsequently assigned as the fentanyl-paired chamber. Mice then underwent 3 consecutive days of conditioning. On conditioning days, mice received an i.p. injection of saline (10 mL/kg) and were confined to the saline-paired compartment for 20 min; 4 h later, they received an i.p. injection of fentanyl (0.2 mg/kg) and were confined to the fentanyl-paired compartment for 20 min. On the fifth day of testing (post-test), after 5 min habituation in the center, they freely explored the entire apparatus for 20 min. CPP score was calculated as the difference in seconds spent on the fentanyl-paired side during post-test vs pre-test. Locomotion during fentanyl conditioning was measured as movement counts in Med-PC V Software.

*Cocaine self-administration*

A cohort of Drd1-cre^120Mxu^ mice underwent intravenous cocaine self-administration as previously described [77]. Briefly, mice underwent 10 consecutive days of cocaine self-administration under FR1 (2 hours/day; 0.5 mg/kg/infusion) using a 10s timeout. Mice then underwent a seeking test 24h after the last self-administration session. This procedure was chosen to facilitate comparison between Drd1-cre^120Mxu^ mice and the unstressed, pair-housed, wildtype mice (purchased from Jax) from our published dataset [77].

*Sucrose self-administration*

Mice were food restricted to maintain 90-95% free-feeding bodyweight starting 4 days prior to the start of sucrose self-administration and lasting throughout the entirety of self-administration. Mice self-administered sucrose on the same schedule and with the same cues as described for fentanyl self-administration above, except sessions lasted 1 hr and sufficient active nose-pokes resulted in delivery of a 20 mg sucrose pellet (chocolate flavor, Bio-Serv #F05301, Flemington, NJ) to a pellet receptacle equipped with infrared head entry detection (ENV-303HDA, MED Associates). Twenty-four hours after the last self-administration session, all mice underwent a 1 hr seeking test under extinction conditions in which responses in the active nose-poke resulted in cue presentations but no sucrose delivery. Immediately after the seeking test, mice were provided with ad-libitum food, then 24h later underwent an additional seeking test. 100% of wildtype mice were littermates from Drd1-cre^120Mxu^ breeding.

*Locomotion*

Mice were injected with 10 mL/kg saline, or 30 mg/kg SKF-38393 s.c., then placed in the center of a Med-Associates ENV-520 open field under dim red light. Infrared beam breaks were recorded for 90 min, in 5 min block intervals, using Activity Monitor Software (Med-Associates SOF-811). Settings for distance traveled, jumps, and stereotypic movement used default software parameters.

*qRT-PCR*

Mice were euthanized by cervical dislocation, and brains were rapidly removed and chilled in ice cold PBS. Cold brains were cut into 1 mm coronal sections using an aluminum brain matrix, and tissue punches containing total NAc or VTA were collected with 14-gauge needle, then snap-frozen on dry ice and stored at -80 until processing. RNA was extracted using Trizol (Invitrogen) and the RNeasy Mini Kit with a DNAse step (Qiagen). RNA concentration was measured on a Nanodrop (ND-8000, Thermo), and cDNA was synthesized with the iScript cDNA synthesis kit (Bio-Rad) using 400 ng of RNA. mRNA expression changes were measured with quantitative polymerase chain reaction (qPCR) with PerfeCTa SYBR Green FastMix (QuantaBio). Fold change mRNA was determined using the 2^-ΔΔCt^ method, using *Gapdh* as the housekeeping gene, and experimentally naïve, wildtype mice as the reference group (males and females combined). Primer sequences are in Supplemental Table 1. Wildtype mice were all wildtype littermates from Drd1-cre^120Mxu^ breeding. For gene expression in mice that underwent fentanyl self-administration, we elected to use experimentally naïve wildtype mice as the reference group because of baseline gene expression differences across genotype. This also allowed us to identify gene expression changes as a function of fentanyl-experience in both wildtype and Drd1-cre mice, since using wildtype IVSA mice as the reference group sets their gene expression to 1.

*Viral vectors & Stereotaxic Surgery*

Adeno-associated viruses for Cre recombinase (pENN.AAV.hSyn.HI.eGFP-Cre.WPRE.SV40), Cre-dependent Gq-coupled (AAV9-hSyn-DIO-hM3D(Gq)-mCherry) or Gi-coupled (AAV9-hSyn-DIO-hM4D(Gi)-mCherry) Designer Receptors Exclusively Activated by Designer Drugs (DREADDs), and mCherry control (AAV9-hSyn-DIO-mCherry) were acquired from Addgene (viral preps # 105540-AAVrg, 44361-AAV9, 44362-AAV9, 50459-AAV9). Adeno-associated Cre-switch virus to label Cre positive and negative neurons (AAV9-Ef1a-DO-DIO-TdTomato-EGFP-WPRE, Addgene plasmid # 37120; http://n2t.net/addgene:37120) was obtained from the University of Maryland School of Medicine Viral Vector Core (Baltimore, MD)

Mice were anesthetized with 1-4% isoflurane gas in oxygen delivered at 1 liter/min, and affixed in a stereotaxic frame (Stoelting, Dale IL). A small burr hole was made over the NAc core and/or VTA, using the following coordinates (in mm, relative to bregma): NAc core (AP +1.6, ML +1.5, DV -4.4), VTA (AP -3.2, ML +1.0, DV -4.6). Viral vectors were delivered with Hamilton neurosyringes with 33-gauge needles at a rate of 100 nL/min for a total volume of 300 nL/virus, then needles were left in place for 5 min to minimize spread up the tract. For Drd1-cre^120Mxu^ mice, Cre-dependent DREADDs or Cre-switch viruses were infused bilaterally in NAc. For wildtype mice, retrograde Cre was infused bilaterally in VTA, and Cre-dependent DREADDs infused bilaterally in NAc.

*Immunostaining*

Mice were transcardially perfused with PBS and 4% paraformaldehyde. Brains were removed and post-fixed for 24 hr, then sectioned to a thickness of 50 µm with a compresstome (Precisionary Instruments, Ashland MA.) Slices were washed with PBS and blocked for 30 min in in PBS with 3% normal donkey serum (NDS) and 0.3% Triton X-100. Slices were incubated at 4ºC overnight in blocking buffer containing appropriate primary antibodies (1:1000, chicken anti-GFP Aves Lab, Tigard, OR, #GFP-1020, Guinea Pig anti-RFP, Synaptic Systems, Goettingen, Germany #390004). Slices were washed 3 x 10 min with PBS, then incubated for 2h in PBS containing secondary antibodies (1:500, Goat anti-Chicken-FITC, Aves Lab, #F-1005; Donkey Anti-Guinea Pig-TRITC, Jackson Immuno, West Grove, PA, # 706-025-148). Slices were washed with PBS 3 x 10 min, mounted with Fluoromount-G (Southern Biotech, Birmingham, AL) and imaged on a laser-scanning confocal (Leica SP8) or epifluorescence microscope (Nikon Labophot2). Sections from mice expressing Cre-switch DO-tdTom-DIO-eGFP virus were counterstained with DAPI.

*D1 vs D2 cell counts*

Brains from Drd1-cre^120Mxu^ expressing Cre-switch virus were processed as above (3♀, 2♂). Three NAc sections per mouse were imaged at 40x magnification on a confocal microscope. 12-15 images surrounding NAc core per hemisphere were stitched together in Leica software, then split into separate DAPI/GFP and DAPI/RFP maximum intensity projections in FIJI. Number of green (Cre-positive, D1) and red (Cre-negative, D2) cells in each hemisphere were counted by two independent experimenters, and averaged together (<10% variance between counters) in FIJI. The percent of GFP and TdTomato positive cells was calculated by adding up the total number of green cells and red cells across all 6 NAc images per mouse (2 hemispheres/slice x 3 slices). Then a single percent positive GFP or TdTomato value from each mouse was averaged together across all mice to generate mean±sem. No cells expressed both GFP and TdTomato.

*Chemogenetic experiments*

For chemogenetic manipulations during fentanyl seeking, stereotaxic surgery was performed as above 24 h after the last self-administration session, allowing 14d for viral expression before the seeking test. DREADDs were activated via i.p. injection of deschloroclozapine (DCZ, 0.1 mg/kg) 20 min before the seeking test. Mice were perfused after seeking and brains were processed for histological verification of DREADDs expression in NAc using above procedures. For chemogenetic manipulation during fentanyl CPP, stereotaxic surgery was performed on naïve Drd1-cre^120Mxu^ mice, and they began CPP 14d later. Mice were injected with saline (or DCZ) 20 min before the post-test, then 4h later, injected with DCZ (or saline) 20 min before a second post-test. To capture c-Fos mRNA expression resulting from DREADDs activation, a subset of mice were euthanized via cervical dislocation immediately following the second post-test while DCZ was still on board. NAc and VTA punches were collected as above from mice with bilateral mCherry expression, visualized with a stereomicroscope equipped with a fluorescence adapter (NightSea, Hatfield PA).


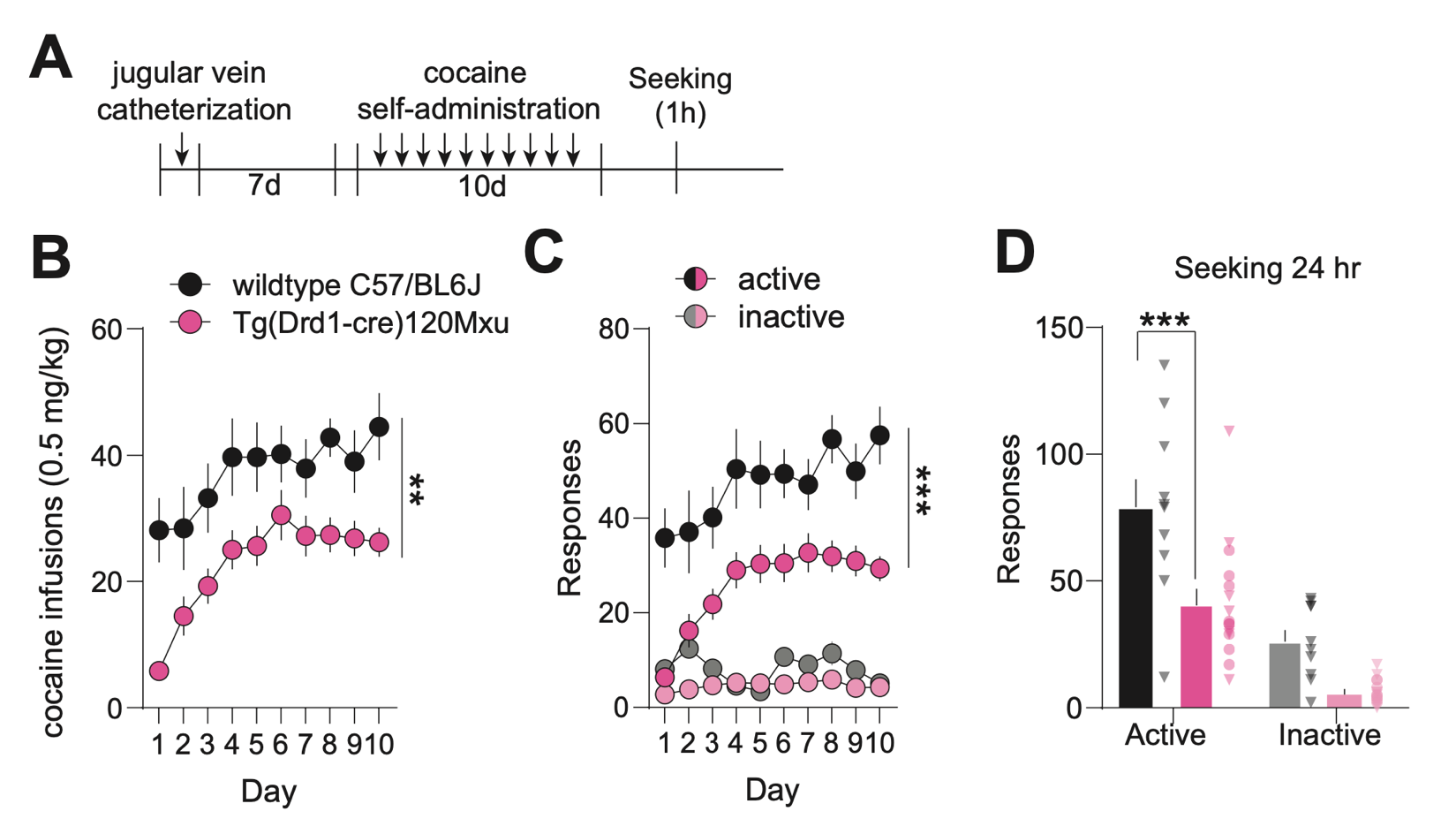


**Supplemental Figure 1. Drd1-cre^120Mxu^** **mice exhibit reduced cocaine self-administration and seeking** (**A**) Experimental timeline for cocaine self-administration experiments. Following recovery from jugular catheterization surgery, mice underwent 10 days of cocaine self-administration training (0.5 mg/kg/inf) under FR1 as in our published work [77] **(B**) Number of cocaine infusions earned during self-administration training in wildtype (black) and Drd1-cre^120Mxu^ (magenta) mice. **, p=0.0031, main effect of genotype in RM-ANOVA. (wildtype n=10♂; Drd1-cre^120Mxu^ n~~=~~9♀, 7♂) (**C**) Number of active and inactive responses during cocaine self-administration training in wildtype and Drd1-cre^120Mxu^ mice. ***, p=0.0003, main effect of genotype. (**D**) Number of active and inactive responses during a non-reinforced seeking task 24 hr after the last self-administration session. ***, p=0.0001, Sidak’s post-hoc after 2-way ANOVA.

**
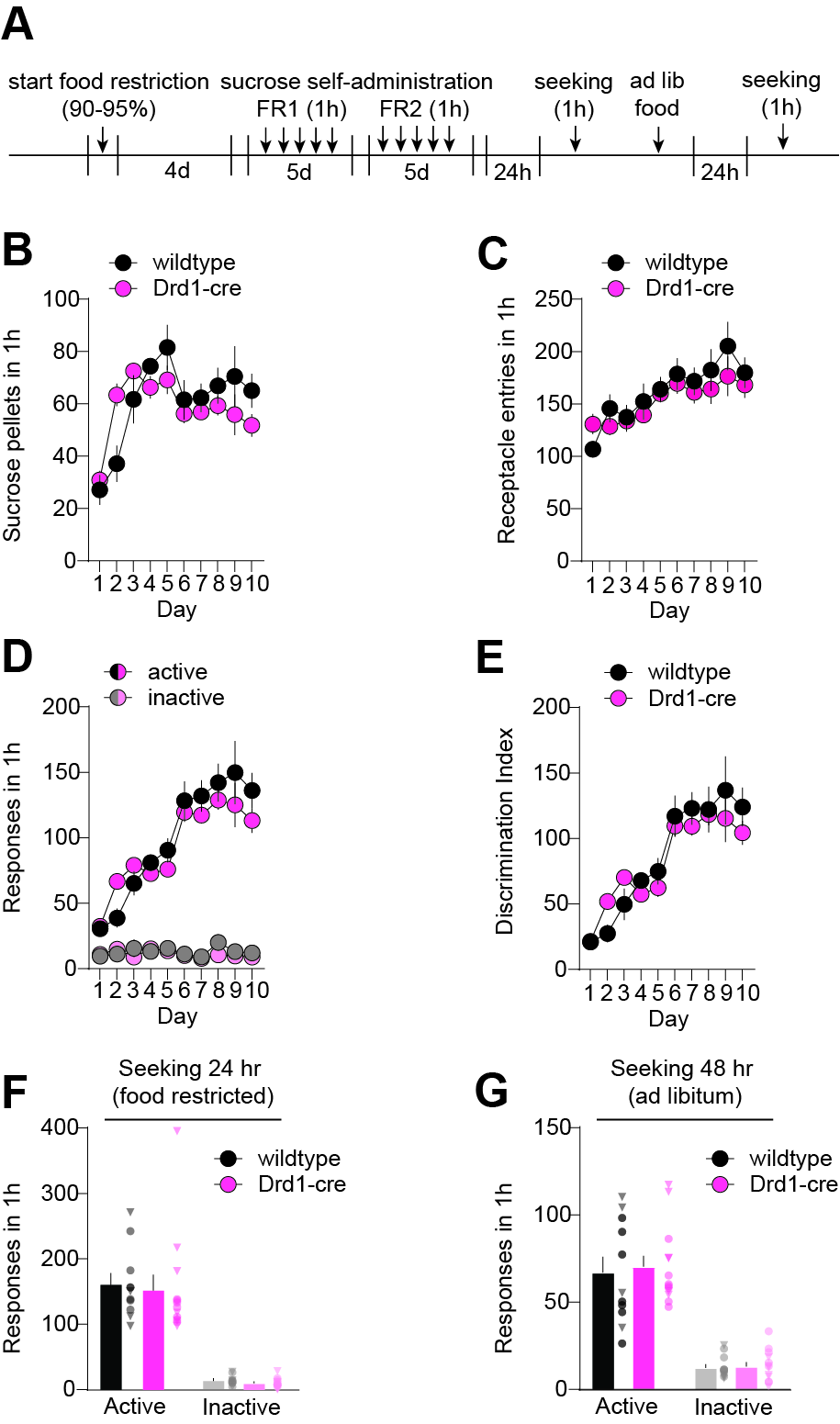
**

**Supplemental Figure 2. Operant sucrose self-administration and seeking do not differ between Drd1-cre^120Mxu^** **and wildtype mice** (**A**) Experimental timeline for sucrose self-administration experiments. Mice were food-restricted to 90-95% of free-feeding bodyweight prior to, and throughout self-administration training. Mice underwent 5 days of sucrose pellet (20 mg) self-administration under FR1, and 5 days under FR2 schedule. Twenty-four hours later, mice underwent a non-reinforced seeking test, were returned to *ad libitum* food overnight, then underwent a second non-reinforced seeking test at 100% free-feeding bodyweight. (**B**) Number of sucrose pellets earned and (**C**) head-entries into the food receptacle in wildtype (black) and Drd1-cre^120Mxu^ (magenta) mice (wildtype n=7♀, 4♂; Drd1-cre^120Mxu^ n=6♀,7♂), (**D**) Responses on the active and inactive nose-poke and (**E**) discrimination index. (**F**) Number of active and inactive responses during a non-reinforced seeking task under food restriction and (**G**) under *ad libitum* food conditions.

**
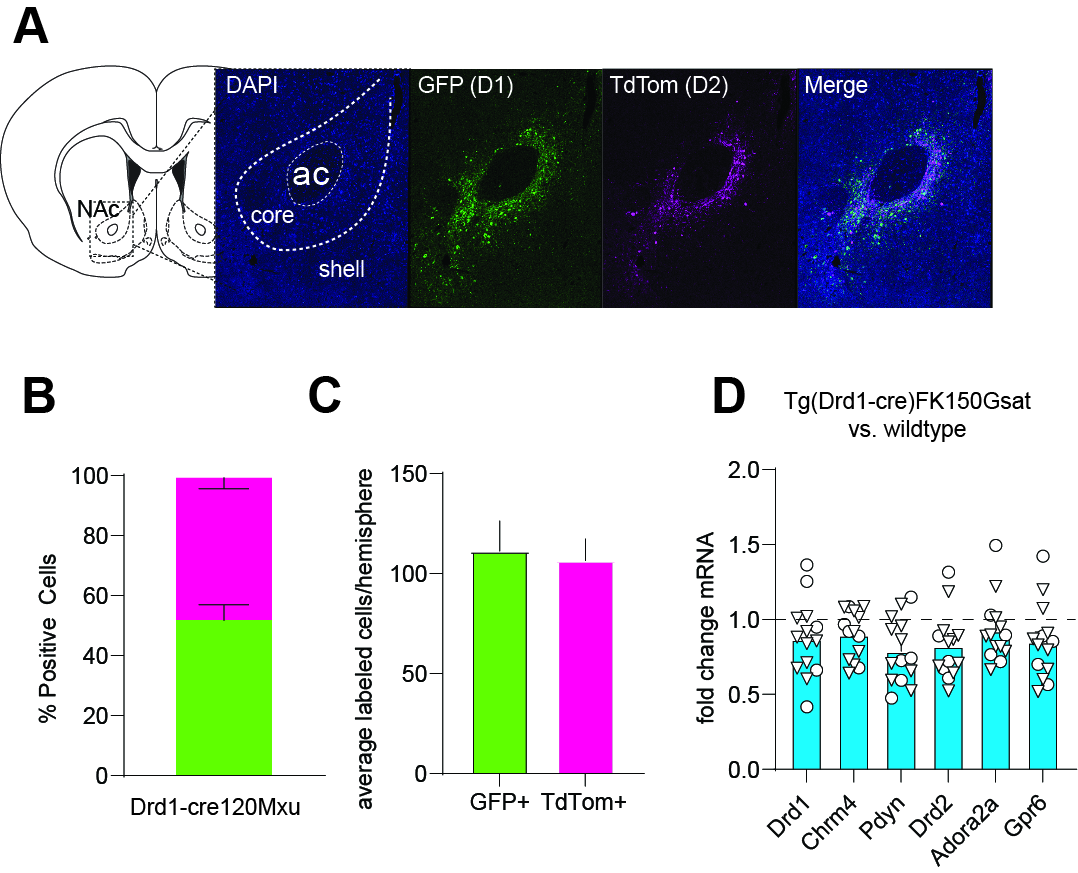
**

**Supplemental Figure 3.** **Drd1-cre^120Mxu^ do not have an imbalance in number of D1:D2 neurons, but uniquely have Drd1 upregulation compared to the Drd1-cre^FK150^ line.** (**A**) Representative image of Cre-switch virus (AAV-DO-TdTomato-DIO-eGFP) expression in NAc core of Drd1-cre^120Mxu^ mice. Cre positive MSNs (D1) express GFP, while Cre-negative (D2) express TdTomato. (**B**) Percent of GFP positive and TdTomato positive cells and (**C**) average number of GFP-labeled and TdTomato-labeled cells per hemisphere across 6 images per mouse (3 sections/mouse, n= 3♀, 2♂) (**D**) Expression of D1- and D2-MSN markers in NAc of a different Drd1-cre line (Gensat FK150, n=5♀, 9♂). Hashed line is average expression wildtype mice (male and female combined). Data are presented as mean±SEM. ac, anterior commissure.


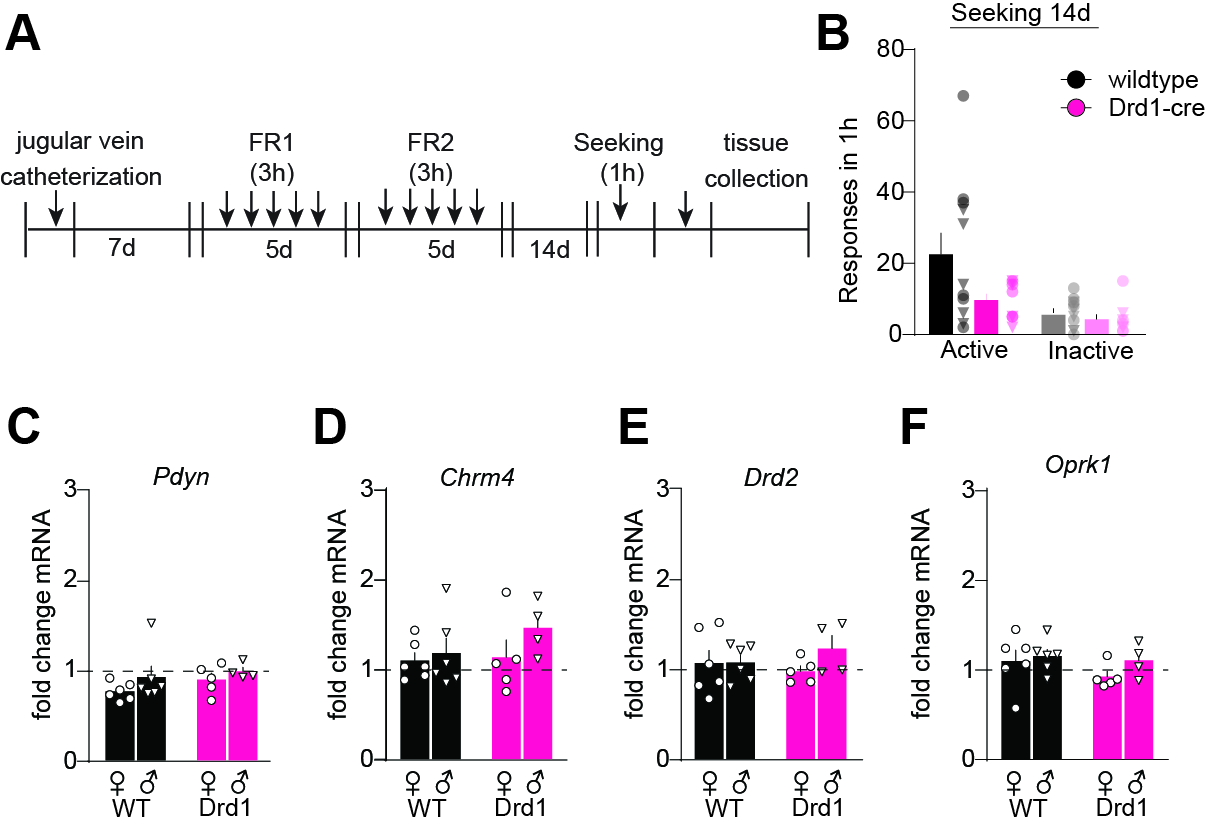


**Supplemental Figure 4. Genes that are not different between Drd1-cre^120Mxu^ and wildtype mice following fentanyl self-administration.** (**A**) Experimental timeline. (**B**) Active and inactive responses during fentanyl seeking in the qPCR subset. (**C-F**) Fold change mRNA relative to experimentally naïve male and female wildtype mice (dashed line; wildtype n=6♀, 6♂; Drd1-cre^120Mxu^ n=5♀, 4♂). Data are presented as mean±SEM. (**C**) Preprodynorphin. (**D**) Muscarinic Acetylcholine receptor M4. (**E**) Dopamine receptor D2. (**F**) Kappa opioid receptor.


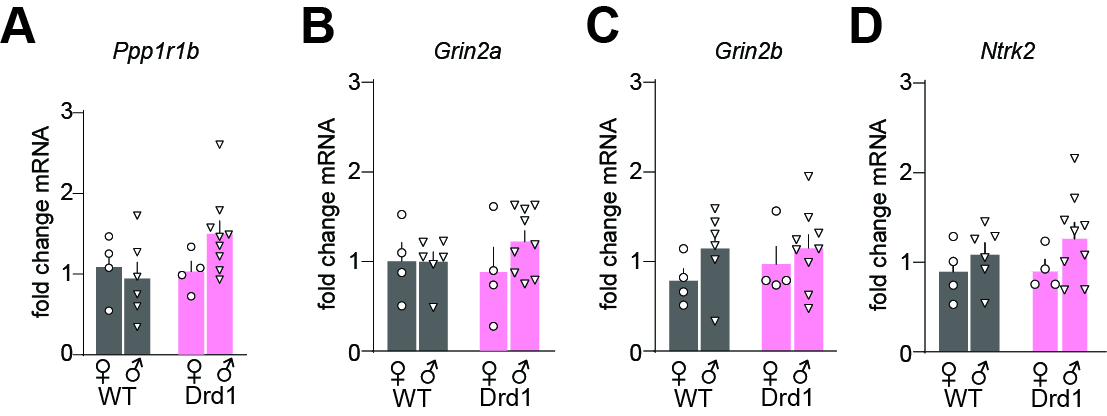


**Supplemental Figure 5. Fentanyl-influenced genes unaltered at baseline in Drd1-cre^120Mxu^ vs wildtype mice.** Fold change mRNA in experimentally naïve wildtype (black) and Drd1-cre120Mxu (magenta) mice, relative to average of naïve male and female wildtype mice (wildtype n=4♀, 6♂; Drd1-cre120Mxu n=4♀, 9♂). Data are presented as mean±SEM. (**A**) DARPP-32. (**B**) NMDAR subunit 2a. (**C**) NMDAR subunit 2b. (**D**) TrkB receptor.

| **Gene** | **Forward Primer** | **Reverse Primer** |
| --- | --- | --- |
| Drd1 | GAGCGTGGTCTCCCAGAT | GGATGCTGCCTCTTCTTCTG |
| Chrm4 | ATCGGCTACTGGCTCTGCTA | TACTGGCACAGCAAAAGGTG |
| Pdyn | CTCCTCGTGATGCCCTCTAAT | AGGGAGCAAATCAGGGGGT |
| Tac1 | TGTTGGACTAATGGGCAAAA | GATAGTGCGTTCAGGGGTTT |
| Drd2 | TCAGATGCTTGCCATTGTTC | GTGAAGGCGCTGTAGAGGAC |
| Adora2a | CACGCAGAGTTCCATCTTCA | AATGACAGCACCCAGCAAAT |
| Gpr6 | GAGGATAGCCAGGCACACAG | ACCACTTGGGACTCGTTGAG |
| Penk | GAGAGCACCAACAATGACGAA | TCTTCTGGTAGTCCATCCACC |
| Oprm1 | CCAGGGAACATCAGCGACTG | GTTGCCATCAACGTGGGAC |
| ­­­Oprd1 | CCATCACCGCGCTCTACTC | GTACTTGGCGCTCTGGAAGG |
| Oprk1 | TCCCCAACTGGGCAGAATC | GACAGCGGTGATGATAACAGG |
| Oprl1 | CGTGCCCTTGATGTTCGGA | GGCCCCAATAGTCCTGAGG |
| Ppp1r1b | CCAACCCCTGCCATGCTTT | TTGGGTCTCTTCGACTTTGGG |
| Ntrk2 | TTGTGTGGCAGAAAACCTTG | ACAGTGAATGGAATGCACCA |
| Grin2a | ACGTGACAGAACGCGAACTT | TCAGTGCGGTTCATCAATAAC |
| Grin2b | GCCATGAACGAGACTGACCC | GCTTCCTGGTCCGTGTCATC |
| Gapdh | AGGTCGGTGTGAACGGATTTG | TGTAGACCATGTAGTTGAGGTCA |

**Supplemental Table 1.** Primer Sequences for qRT-PCR.
